# Stability, seepage and displacement characteristics of heterogeneous branched-preformed particle gels for enhanced oil recovery†

**DOI:** 10.1039/c7ra13152f

**Published:** 2018-01-29

**Authors:** Jiangbo Li, Zuming Jiang, Yi Wang, Jing Zheng, Guangsu Huang

**Affiliations:** College of Polymer Science and Engineering, Sichuan University Chengdu 610041 Sichuan China Guangsu-huang@hotmail.com zhengjing@scu.edu.cn; Research Institute of Exploration & Development, Shengli Oilfield Dongying 257015 China

## Abstract

Inspired by the viscoelastic displacement theory and the advantages of preformed particle gels, we develop an innovative product called branched-preformed particle gel (B-PPG) for enhanced oil recovery. Due to its excellent viscoelastic properties, B-PPG can be used both in profile control and to improve sweep efficiency in heterogeneous reservoirs. Laboratory experiments indicate that B-PPG shows improved stability and long-term aging resistance under high temperature and salinity when compared with HPAM. The migration and displacement behaviors of B-PPG are studied by a series of sandpack core flow experiments. The results show that the B-PPG particles can migrate through the porous media, and the migration is a dynamic process of plugging and flooding. Besides, B-PPG can significantly create fluid diversion and increase the swept volume in low permeability zones. Moreover, micro visualization and oil displacement experiments are also carried out and prove that B-PPG can displace residual oil in small channels, leading to a high swept volume and enhanced displacement efficiency.

## Abstract 2

Inspired by the viscoelastic displacement theory and the advantages of preformed particle gels, we develop an innovative product called branched-preformed particle gel (B-PPG) for enhanced oil recovery.

## Graphical abstract

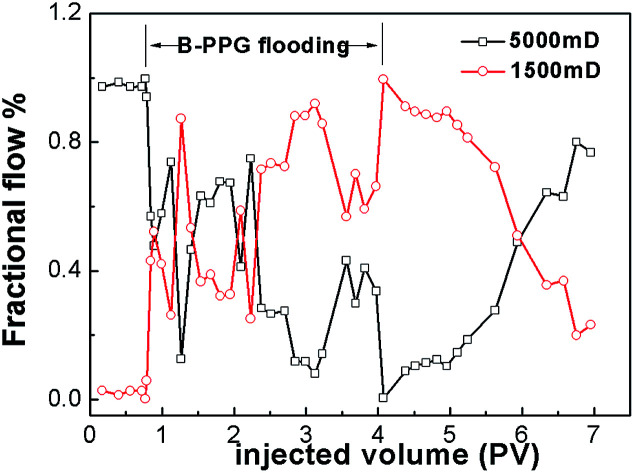

## 1. Introduction

Fossil fuels, such as oil and gas, currently supply most of the world's energy needs and will remain the dominant energy source for at least the next decade or so.^1,2^ At present, large fractions of the oil reserves in the world are located in mature oilfields whose production is declining. Meanwhile, with today's technology, the worldwide average recovery factor for mature reservoirs is in the range of only 30–40%. Enhanced oil recovery (EOR) is believed to be a very effective method to increase the oil recovery and final recovery factors in reservoirs.^3–5^ In addition, polymer flooding is considered one of the most promising technologies in EOR processes due to its technical and commercial feasibility. To date, polymer flooding has contributed to 10% of the original oil in place (OOIP) after water flooding.^6^

In most oil reservoirs, there are many layers of different permeability. Even a single layer can have a heterogeneous composition with numerous preponderance flow paths.^7,8^ Injected water will prefer to flow along the higher permeability layer and the preponderance flow path. As a result, there is still a high amount of remaining oil in lower permeability areas, and oil recovery is very low. One strategy to enhance oil recovery in heterogeneous reservoirs is to improve the sweep efficiency of the lower permeability areas. Usually, a viscous polymer solution is injected as the driving fluid, which would increase the volumetric sweep efficiency. In practice, two commercial polymers, hydrolyzed polyacrylamides (HPAM) and xamthan gums, are mostly used.^9^ More recently, it has been recognized that the elasticity of the polymer solution also has a great impact on improving the recovery efficiency. The elastic stress causes the protruding portion of residual oil to change shape and move.^10^ Research shows that high elastic polymer solutions can result in higher sweep efficiency and lower residual oil saturation compared with low elastic polymer solutions.^11,12^ Thus, a new viscoelastic displacement theory is put forward: the higher the viscoelasticity of the fluid, the higher the oil recovery.^13–15^ The individual contributions of the viscous and the elastic components of the injected polymer have been investigated by Wei.^16^

Another strategy is to correct the sharp difference in permeability that exists in the different formation layers to create a homogeneous reservoir. This approach is termed profile modification or permeability modification.^17^ In recent years, polymer microgels, including colloid dispersed gels, preformed particle gels (PPG), bright water and pH-sensitive cross-linked polymers, have been developed for these modifications. Among those microgels, preformed particle gels seem to be more distinguishable and reliable than the others. They are prepared by using an initiator, acrylamide monomer, a cross-linker and an additive. These powdered micrometer- or millimeter-sized gel particles can be obtained from crushing and then sieving dry gels, which can swell several folds in water to form a suspension. These preformed particle gels can tolerate high salinities and high temperatures and may improve the development of oilfields that exhibit strong heterogeneity, high water-cut, and large porous channels.^18–20^ However, despite the tremendous advantages that preformed particle gels present towards conformance control and reservoir heterogeneity, they seem incapable to improve the sweep efficiency of lower permeability areas because of their low viscosity. Meanwhile, these suspensions are heterogeneous, and the particles are easily subsided, which greatly impacts their injection and migration in channels.

Inspired by the viscoelastic displacement theory and the advantages of preformed particle gels, we propose an innovative product called branched-preformed particle gel (B-PPG), which combines the excellent high viscosity characteristic of linear polyacrylamide with the outstanding profile control ability of PPG. Tough this type of product has already been experimented in the Shengli Oilfield, its ability to produce similar products and to enhance oil recovery has not been reported. Herein, laboratory experiments are performed to characterize the viscoelastic properties, stability and aging resistance of B-PPG under high temperature and salinity. The microscopic images of B-PPG during aging are obtained by environmental scanning electron microscopy (ESEM). Using sand pack models, the migration and displacement mechanism of B-PPG is investigated. In addition, the flooding behavior of PPG in heterogeneous media is further observed by double-core sand pack tests. Moreover, microscopic visual displacement experiments are conducted to test the oil displacement capability of B-PPG. On the basis of these results, B-PPG can not only overcome the prior limitations of linear polymers, such as poor long-term thermal stability, poor salt resistance and low elasticity, but also improve the viscosity, suspension stability and migration ability of PPG. We believe that this novel product can be used as a new oil-displacing agent both to improve sweep efficiency and control the heterogeneous profile.

## 2. Experimental section

### 2.1 Materials

Acrylamide (AM, Chengdu Kelong Chemical Reagent Factory, China, AR), dimethylaminoethyl methacrylate (DA, J&K Chemical, China), potassium persulfate (KPS, Tianjin Bodi chemical industry LTD, China, AR), sodium bisulfite, (NaHSO_3_, Tianjin Bodi chemical industry LTD, China, AR), sodium chloride (NaCl, Tianjin Bodi chemical industry LTD, China, AR), magnesium chloride (MgCl_2_·6H_2_O, Tianjin Bodi chemical industry LTD, China, AR), and calcium chloride (CaCl_2_, Tianjin Bodi chemical industry LTD, China, AR) are used as received. Water used in this study is ion-exchanged water unless otherwise mentioned. Both the partial hydrolyzed linear polyacrylamide (HPAM, *M*_w_ is about 2000W Da) and the commercial preformed particle gel (PPG) are kindly provided by Shengli Oilfield in China. The branched-preformed particle gel (B-PPG) is prepared by free radical polymerization of AM using DA as cross-linking agent under a redox system at room temperature. The detailed synthesis is reported in our previous paper.^21^ Six B-PPG samples with different cross-linking degrees (Table 1) are prepared and used for the tests. More detailed structural information about the six B-PPG samples is given in the ESI.† Brine water of different salinity is prepared according to Table 2. A simulated oil sample with viscosity of 16 mPa s and density of 942.7 kg m^−3^ at 85 °C is prepared using degassed crude oil from Block Gudao in the Shengli Oilfield in China mixed with a certain proportion of kerosene.

**Table 1:** Rheological properties of the six B-PPG samples in brine water (30 000 mg L^−1^)

Sample^a^	*G*′/Pa	*G*′′/Pa
1#	1.206	1.323
2#	4.053	1.672
3#	6.206	1.987
4#	7.705	2.877
5#	9.449	2.716
6#	11.989	2.319

a The modulus of all the B-PPG samples is measured by dynamic oscillation measurements on the rheology: the gap is 200 μm, frequency is 1 Hz, and the stress is 0.1 Pa.

**Table 2:** Composition of the saline solutions used

Mineralization	H_2_O	NaCl	CaCl_2_	MgCl_2_·6H_2_O	Na_2_SO_4_
6666 mg L^−1^	1000 mL	6.191 g	0.241 g	0.3514 g	0.0696 g
19 334 mg L^−1^	1000 mL	17.457 g	1.143 g	0.863 g	0
30 000 mg L^−1^	1000 mL	27.306 g	1.11 g	3.833 g	0
50 000 mg L^−1^	1000 mL	42.758 g	2.825 g	8.917 g	0

### 2.2 Viscoelastic measurements

The viscoelastic properties of the B-PPG suspensions are measured with a rheometer (AR2000ex, TA instrument Corp., USA) equipped with a Peltier device for temperature control in which a 40 mm stainless steel upper plate is used as clamping fixture. The viscosity is measured under state shearing modes where the gap is 1000 μm. The shearing rate rage is 2–100 s^−1^. The dynamic modulus (storage modulus *G*′ and loss modulus *G*′′) is measured under dynamic oscillation modes. The maximum *G*′ is obtained, corresponding to a gap of 200 μm (Fig. S4†). The frequency range is 0.01–10 Hz, and the stress is 0.1 Pa. All the measurements are conducted at 85 °C.

### 2.3 Thermostability tests

To determine the long-term high temperature tolerance of the B-PPG suspension, aging tests are conducted at 85 °C. For each sample, 1 g of the dry particle gels are measured and dissolved in 200 mL brine water (30 000 mg L^−1^). Then, the suspension is divided into 10 parts, and each is poured into one ampoule. Next, the ampoule is vacuumed to remove any oxygen in the suspension and is later bubbled with N_2_. At last, the ampoules are sealed and placed into the aging oven. After specific aging times, each ampoule is taken out of the oven and cooled to room temperature. Then, the viscosity and elasticity of the B-PPG suspension is monitored with the rheometer.

### 2.4 Environmental scanning electronic microscopy (ESEM)

To observe the microstructure of B-PPG and PAM during the aging tests in water, a quanta 450 environmental scanning electronic microscope (ESEM) (American FEI Company) is utilized, with an annular gaseous secondary electron detector (GSED) at a low vacuum model. The concentration is 1000 mg L^−1^. 0.1 mL of the suspension or solution is dropped into a stainless-steel concave cell which is sealed to prevent solvent evaporation. The sample is cooled to −10 to −15 °C within 1 minute at room temperature using a Peltier electric heating and cooling device. Once the sample is totally frozen, it is immediately transferred into the sample room of the electron microscope. The vacuum degree and temperature are adjusted to sublimate the ice, and finally the polymer in suspension or solution is left.

### 2.5 Single-core sand pack experiment

**Fig. 1:**
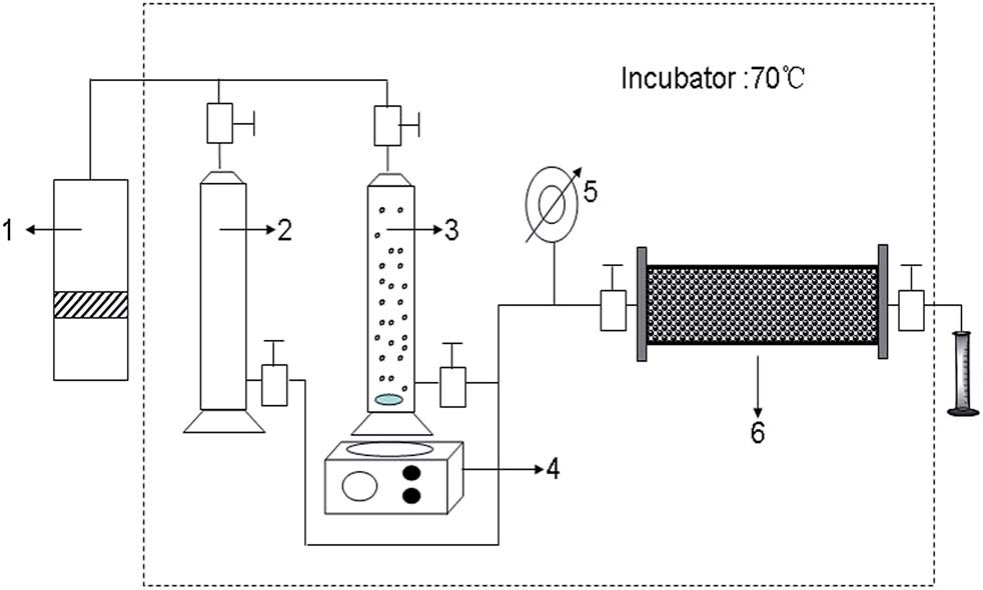
Schematic diagram of the core flow experimental apparatus (1: pump 2: saline solution 3: B-PPG suspension 4: magnetic stirrer 5: pressure gauge 6: sand packed core model).

The main equipment used is the experiment flow for chemical flooding, as shown in Fig. 1. The size of the sand pack is 2.5 cm in diameter and 30 cm in length. Sand (Block Gudao in the Shengli Oilfield, China) is gradually packed into the model with a constant packing pressure (200 psi) to ensure that all sections of the sand pack have the same porosity. The sand pack flooding tests are conducted horizontally. Each sand pack is first injected with brine water (30 000 mg L^−1^). Meanwhile, the inlet pressure is monitored at regular intervals. When the equilibrium pressure is reached, the B-PPG suspension (2000 mg L^−1^) is injected. After the pressure is balanced, water flooding is conducted again until the pressure change is negligible. The injection rate is 0.5 mL min^−1^, and the temperature is 85 °C.

### 2.6 Two-step core sand pack test

**Fig. 2:**
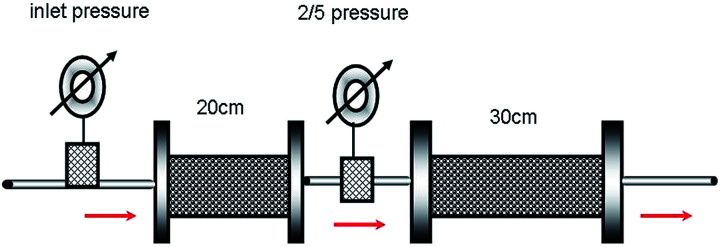
Schematic of the series connection of the two-step core sand pack experiment.

The procedure followed is the same as that of the single-core sand pack experiment except that two sand packs are used. The two sand packs have the same permeability (1500 ± 15 × 10^−3^ μm^2^) but different lengths (one is 20 cm long and the other is 30 cm long) and are connected together (see Fig. 2). Two pressure gages are placed at the entrance and joint to measure the inlet pressure and the 2/5 pressure, respectively.

### 2.7 Double-core sand pack test

**Fig. 3:**
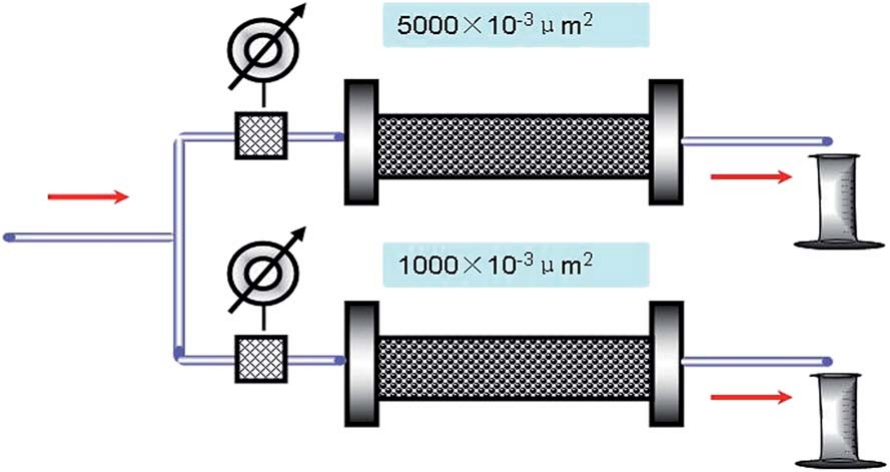
Schematic of the parallel connection of the double tube core flow experiment.

The procedure followed is also the same as that of the single-core sand pack experiment except that two sand packs are used. The two sand packs have different permeabilities (one of (1000 ± 10) × 10^−3^ μm^2^ and the other of (5000 ± 15) × 10^−3^ μm^2^) and are arranged in parallel (see Fig. 3). Two amounts of 1 PV brine water (30 000 mg L^−1^) are injected consecutively. Then, water flooding is conducted again. The diversion flow volumes for the high- and low-permeability sand packs are measured at a given time interval.

### 2.8 Micromodel test

**Fig. 4:**
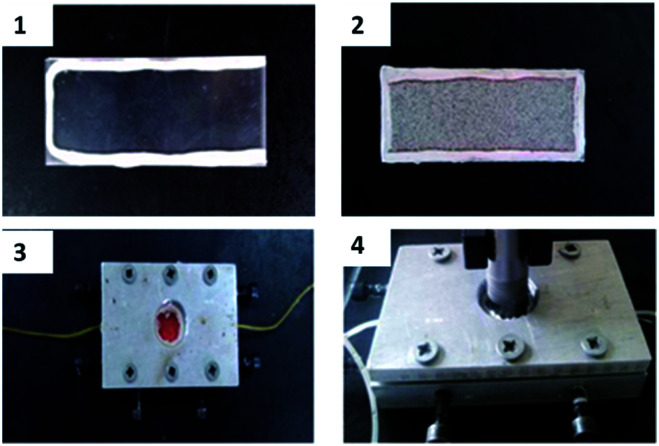
Preparation of the model for microscopic oil displacement.

The autonomous micromodel using glass beads used for the micromodel tests is shown in Fig. 4. First, two smooth acrylic plates are stuck together by daub, and two syringe needles are inserted in the two short sides. Then glass beads are used to compactly fill the two plates. Afterwards, the plates are put in a model equipped with a HD video camera. The experiment procedure is as follows: (1) the micromodel is saturated with brine water (30 000 mg L^−1^); (2) the micromodel is saturated with simulated oil; (3) brine water is slowly injected in the micromodel until no oil is produced from the outlet; (4) the HPAM solution is injected at the same rate until no oil is produced from the outlet; (5) the B-PPG suspension is injected at the same rate until the oil production becomes negligible. The dynamic images of the oil displacement process are captured by the camera throughout the experiments. Another micromodel test is conducted with the microscopic displacement physical simulation system (Haian petroleum scientific instrument Corp., China), which is equipped with a high-power microscope and an etched glass model. The etched glass model can help observe the seepage of oil and water in the model and the distribution of residual oil by oil flooding, water flooding, polymer flooding and other chemical flooding. The experimental procedure is the same as above.

### 2.9 Oil displacement test

The laboratory oil displacement experiment is carried out as follows. The wet-packed sand pack is flooded with the oil sample until the water production ceases.

Thereafter, brine flooding is continued until the water cut is greater than 98%. After that, a 0.3 pore volume (PV) of the HPAM solution or B-PPG suspension is injected (2000 mg L^−1^); this is followed by water flooding until the water cut of the efflux reaches 98% again.

## 3. Results and discussion

### 3.1 Viscoelastic properties of B-PPG

**Fig. 5:**
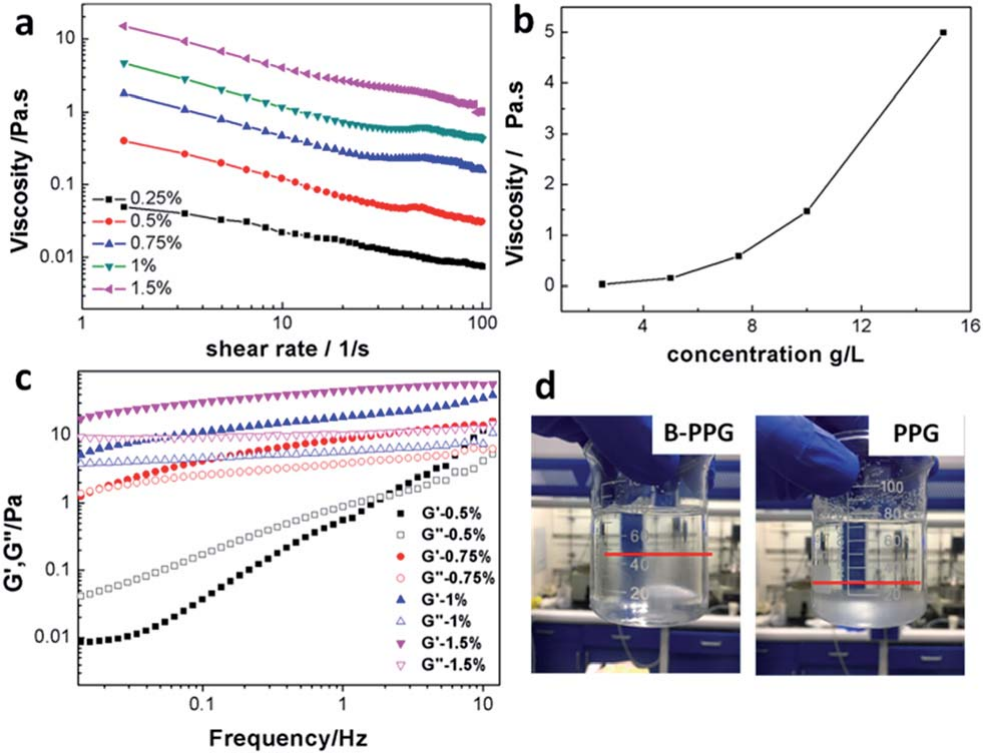
Viscosity *versus* shear rates of B-PPG (sample #1 is used) at different concentrations with a salinity of 30 000 mg L^−1^ at 25 °C (a); viscosity *versus* concentration at the shear rate of 7.34 1/*s* (b); variation of the dynamic modulus (*G*′, *G*′′) of B-PPG *versus* frequency at different concentrations (c); suspension behavior of B-PPG and PPG in brine water (d).

Fig. 5a plots the viscosity *versus* shear rates of B-PPG in brine water with a salinity of 30 000 mg L^−1^ at 25 °C, and Fig. 5b shows the viscosity *versus* concentration at the shear rate of 7.34 1/*s*. For the B-PPG suspension, the apparent viscosity increases with an increasing concentration of B-PPG. High concentrations cause the volume fraction of B-PPG in water to increase as well as the entanglement of branched chains, and thus the apparent viscosity increases. The viscosity at a concentration of 5 g L^−1^ is about 145 mPa s, which is much higher than that of commercial preformed particle gels. Due to their high viscosity, these particle gels can suspend in the brine water for a long time with a fairly large suspension volume (above 60%), while for the traditional PPGs, they subside soon (Fig. 5d).

The viscoelasticity of B-PPG is observed by measuring its dynamic modulus. The dynamic modulus includes two parts, that is, the storage modulus (*G*′) and the loss modulus (*G*′′). The storage modulus represents the energy stored during the deformation process and can be recovered when the stress is removed. It arises from the transient change in network chain conformation and thus is used to characterize 'elasticity'. The loss modulus represents the energy that has been used for the deformation of B-PPG or the internal friction energy. It results from the change in network chain conformation arising from the absorption of external energy and thus can be used to characterize 'viscosity'.^22,23^Fig. 5c shows the variation of the dynamic modulus (*G*′, *G*′′) of B-PPG *versus* frequency at different concentrations. The storage modulus and loss modulus at 1 Hz for a concentration of 1% are 18.61 Pa and 5.7 Pa, respectively, indicating that B-PPG has a good viscoelasticity. Within the frequency range of 0.01–10 Hz, both *G*′ and *G*′′ increase as the frequency increases. In addition, the increase trend slows down as the concentration increases. The higher the concentration, the greater the modulus value and the more significant the characteristics of the viscoelasticity. An intersection of *G*′ and *G*′′ can be observed at concentrations of 0.5% and 0.75%, which means that the viscoelastic behavior of the B-PPG suspension changes from viscosity-dominant to elasticity-dominant. When the concentration is higher, no crossover is generated, and *G*′ is always dominant. This observation can be interpreted as follows: as the concentration increases, the entanglement between branched chains is improved. As a result, the cross-linked network is more intensive, which causes elasticity to be dominant at low frequency.

The viscoelastic properties reflect the elastic nature of the B-PPG, which can deform to change its shape because of the drag force caused by water flow. The deformed B-PPG particles can recover their original shape and size after leaving the small pore-throats for the large pores. Meanwhile, the suspension of B-PPG also has considerable viscosity. It is expected that B-PPG can be used both in dynamic profile control and to improve sweep efficiency in heterogeneous reservoirs.

### 3.2 Improved stability of B-PPG under high temperature and salinity

**Fig. 6:**
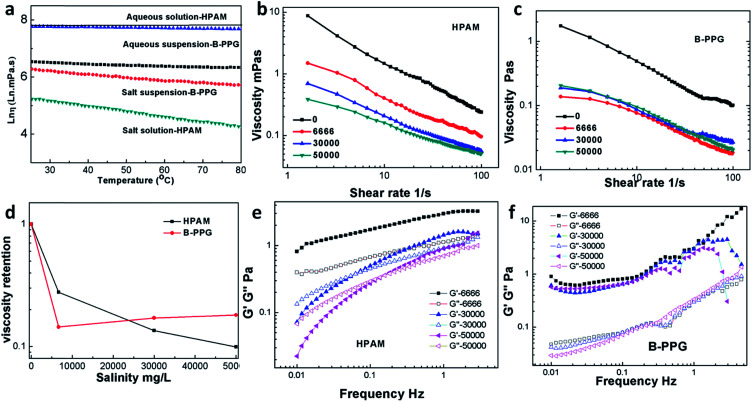
Shear viscosity of B-PPG (sample 1# is used) and HPAM *versus* temperature in aqueous and salt solutions (a); shear viscosity of HPAM (b) and B-PPG (c) in different salinities; viscosity of HPAM and B-PPG *versus* salinity at a shear rate of 7.34 1/*s* (d); modulus of HPAM (e) and B-PPG (f) in different salinities.

The stability of the B-PPG suspension is evaluated by measuring the viscosity under different temperatures and salinities. For the purpose of comparison, a solution of linear, partially hydrolyzed polyacrylamide (HPAM, with the same concentration) is also measured. Fig. 6a shows the shear viscosity *versus* temperature in aqueous and salt solutions. For both products, the viscosity is almost constant in aqueous solution but varies significantly with temperature in a saline solution. Their relationship can be fitted by Arrhenius' eqn (1) where the flow activation energy (*E*_a_) can be calculated and used to describe the dependence of viscosity on temperature.^24^1
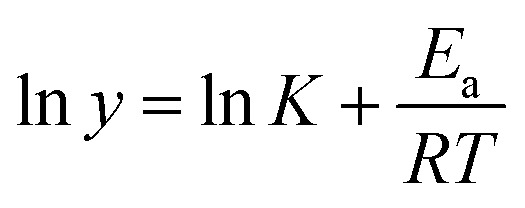
The *E*_a_ of B-PPG in salt solution is much lower than that of HPAM (Table 3), which means B-PPG has a low temperature sensitivity.

**Table 3:** *E*_a_ values of B-PPG and HPAM

Polymer	*E*_a_ (KJ mol^−1^)	ln *K*	Increase of *E*_a_
B-PPG salt solution	8.843	3.398	2.737
HPAM salt solution	15.993	−1.171	11.497
B-PPG aqueous solution	3.231	5.543	—
HPAM aqueous solution	1.391	7.532	—

Additionally, the viscosity and modulus under different salinities are also investigated. When the brine concentration increases from 0 to 6666 mg L^−1^, the solution viscosity of both B-PPG and HPAM decreases sharply (Fig. 6b and c), and no precipitation and phase separation behavior is observed. For higher salinities, the solution viscosity of HPAM decreases slowly but barely changes for B-PPG. Generally, the thickening capability of HPAM arises from hydrodynamic chain entanglement and electrostatic repulsion.^25^ The presence of electrostatic charges in solution results in a decrease in electrostatic repulsion, eventually leading to the decrease of the viscosity. However, the larger branched chains and hydraulic volume of B-PPG cause the stretched polymer chain to be more stable in the presence of monovalent and even divalent ions when compared with HPAM. Meanwhile, although the viscosity of B-PPG is lower than that of HPAM in aqueous water, it is much higher in saline solutions. Fig. 6e and f display the *G*′ and *G*′′ of HPAM and B-PPG at different concentrations of brine solution, respectively. With the increase in salinity, *G*′ and *G*′′ remain almost constant for B-PPG while they decrease for HPAM. This result can indicate that the viscoelasticity of HPAM is governed by chain entanglement, while that of B-PPG originates from the cross-linked structure. Overall, B-PPG possesses a better compatibility with salt than HPAM.

### 3.3 Aging resistance under high temperature and salinity

**Fig. 7:**
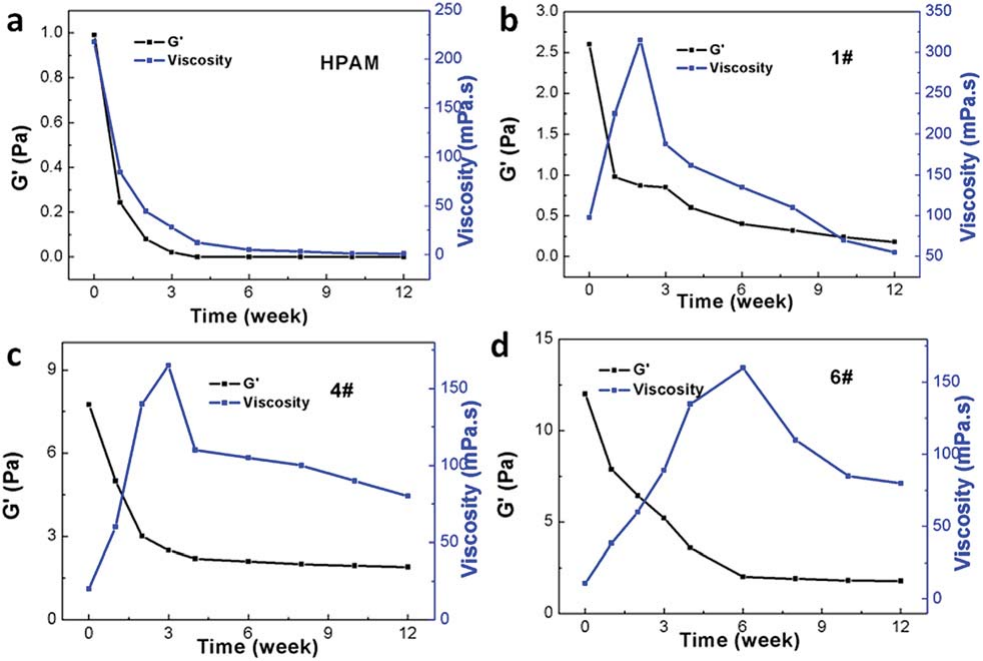
Curves of storage modulus and viscosity with time (representing long-term thermal stability) for HPAM and three types of B-PPG with different crosslinking degree.

Fig. 7 presents the long-term thermal resistance of HPAM and three types of B-PPG with different cross-linking degrees. As is clearly shown in Fig. 7a, the viscosity of HPAM rapidly decreases to a value of 1.1 mPa s within 6 weeks, whereas for B-PPGs, it increases first and then slowly decreases. After 12 weeks, the viscosity of the B-PPG samples still remains higher than 78 mPa s. Additionally, for B-PPG, the higher the cross-linking degree, the longer the viscous increasing stage, indicating an increase in the long-term thermal resistance. Furthermore, the *G*′ of HPAM quickly decreases to 0 Pa in 4 weeks, while the final *G*′ of B-PPG is still higher than 0 Pa after 12 weeks. Such dramatic improvement on the long-term thermal resistance of B-PPG is one key reason why they are potentially valuable for enhanced oil recovery.

### 3.4 Environmental scanning electron microscopy imaging of B-PPG and HPAM during the long-term aging test

**Fig. 8:**
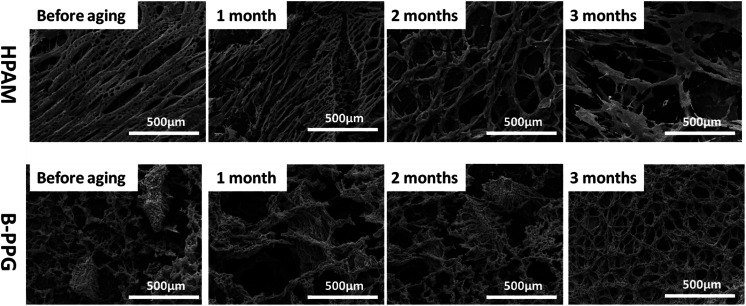
Variation in the ESEM images for HPAM and B-PPG in saline solution during aging tests.

Fig. 8 presents the ESEM micrographs of HPAM and B-PPG during the long-term aging tests. A porous interconnected network structure is observed in HPAM before aging. However, the pores become larger, and the network is looser as the aging time increases. After 3 months, the network is disjointed and completely broken. In contrast, there are a number of insoluble gel particles in the suspension of B-PPG, which is cross-linked. The sizes of these particles are about 300 μm. After one month, the gel particles are fully swollen and slightly larger, with flocs on their surface. This observation is expected because the cross-linking points in B-PPG are broken, and the branched chains are released. After 2 months, these particles can still be observed clearly. A homogenous porous network structure is observed after 3 months, which indicates the degradation of the gel particles into the polymer solution. The degradation behavior of B-PPG is similar to the degradable nanocomposite preformed particle gel reported by Paul Tongwa.^26^ Based on their high post-degradation viscosity, they are recommended for secondary polymer flooding.

### 3.5 Migration and displacement characteristics of B-PPG

**Fig. 9:**
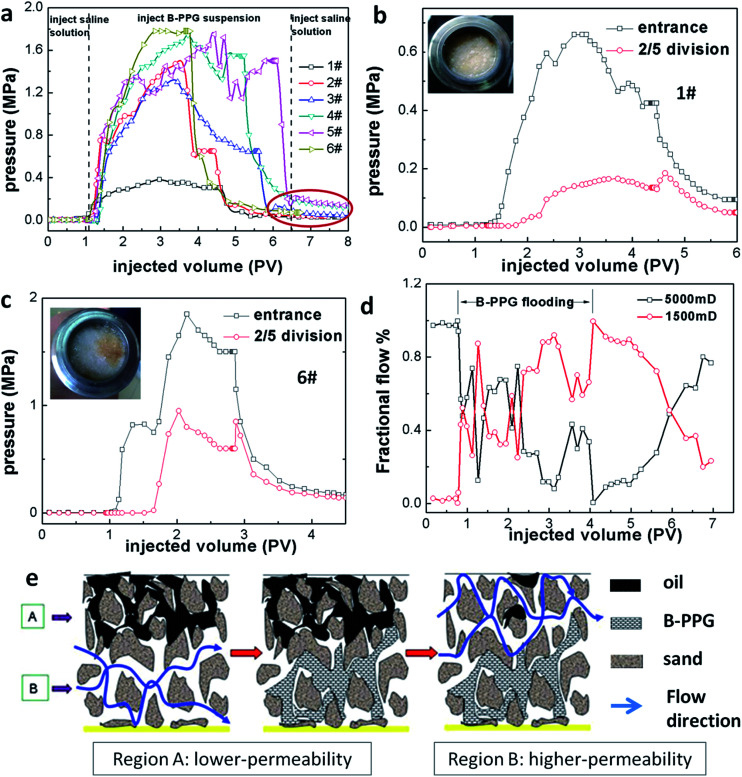
Pressure curves of different B-PPG suspensions during flooding (a); pressure curves at different points of the sand packed core during B-PPG flooding (b and c); fractional flow curve in the parallel double-sand tests during B-PPG (sample 6#) flooding (d); schematic of the fluid diverting mechanism of the B-PPG suspension (e).

Fig. 9a represents the pressure curves of the different samples during flooding. One observes that the curves for B-PPG are fluctuating, which is caused by the combined plugging and flooding of the B-PPG suspension. When the pressure reaches the driving pressure of the B-PPG particles, the particles can change their shape and deform to pass through the pore-throat, resulting in a decrease in the plugging effect and a reduction in the pressure differential. With further injection of B-PPG suspension, the sand packed core is plugged again, the particles again deform to pass through, resulting in the fluctuation in pressure differentials. The phenomenon of fluctuation in pressure differentials indicates that the migration of B-PPG particles through the porous media is a simultaneous plugging and flooding process. When a saline solution is injected, the pressure differential is significantly reduced, which shows that injection of saline solutions can displace B-PPG particles away from their plugging positions. However, the pressure stays at an elevated level, as shown in the red circle, indicating that some pore-throats remain blocked, and B-PPG has good resistance to long-term water flushing. With the increase in cross-linking density, the equilibrium pressure of flooding becomes higher. Thus, the modulus of the B-PPG solution can be used to predict the steady-state pressure differential in core flow experiments.^27^

To further evidence the migration of B-PPG, we conduct a two-step sand core test. A pressure gage is installed between a 20 cm-long and a 30 cm-long sand core. The measured pressure is defined as the 2/5 division pressure. The 2/5 division pressure is proportional to the inlet pressure (Fig. 9b and c) during B-PPG flooding, indicating that the pressure can be well transmitted. In the subsequent water flooding, the inlet pressure immediately drops, while the 2/5 division pressure first increases and then decreases. As soon as water flooding begins, the particles in the previous sand core are washed away, while the particles in the latter sand core does not change. Thus, the pressure is concentrated, causing the pressure to increase. Moreover, after flooding, no filter cake is observed on the cross section of the core. All of these results strongly demonstrate the migration of particles in the sand cores.

### 3.6 Fluid diversion of B-PPG by the double-core sand pack test

Furthermore, the flooding of B-PPG in heterogeneous media is investigated by the double core sand pack tests. Before the injection of the B-PPG suspension, the initial shunt rate of the high- and low-permeability tubes is 83.5% and 16.5%, respectively. The ratio of the initial shunt rates is about 5 : 1, which coincides with the ratio of the permeability. With the injection of the B-PPG suspension, the flow in the low-permeability sand pack increases while it decreases in the high-permeability sand pack, producing an unusual phenomenon called fluid diversion. The same observation was also reported by Bai^28^ when they investigated the profile control properties of preformed particle gels. At the beginning of the injection, the B-PPG particles enter the high-permeability pack first and penetrate into the lower permeability pack when the pressure across the high-permeability pack rises high enough (Fig. 9e). The maximum liquid yield of the high- and low-permeability sand packs are 43.18% and 56.82%, respectively, indicating that B-PPG can effectively adjust the profile of the heterogeneous media. In the extended water flooding stage, the fractional flow of the high-permeability pack increases slowly while it drops in the low-permeability pack. They achieve the same value after 2 PV and gradually return to the fractional flow before B-PPG flooding. This result reveals that B-PPG injection can significantly create fluid diversion and increase the swept volume in low permeability zones.

### 3.7 Microscopic visualization and oil displacement experiment

**Fig. 10:**
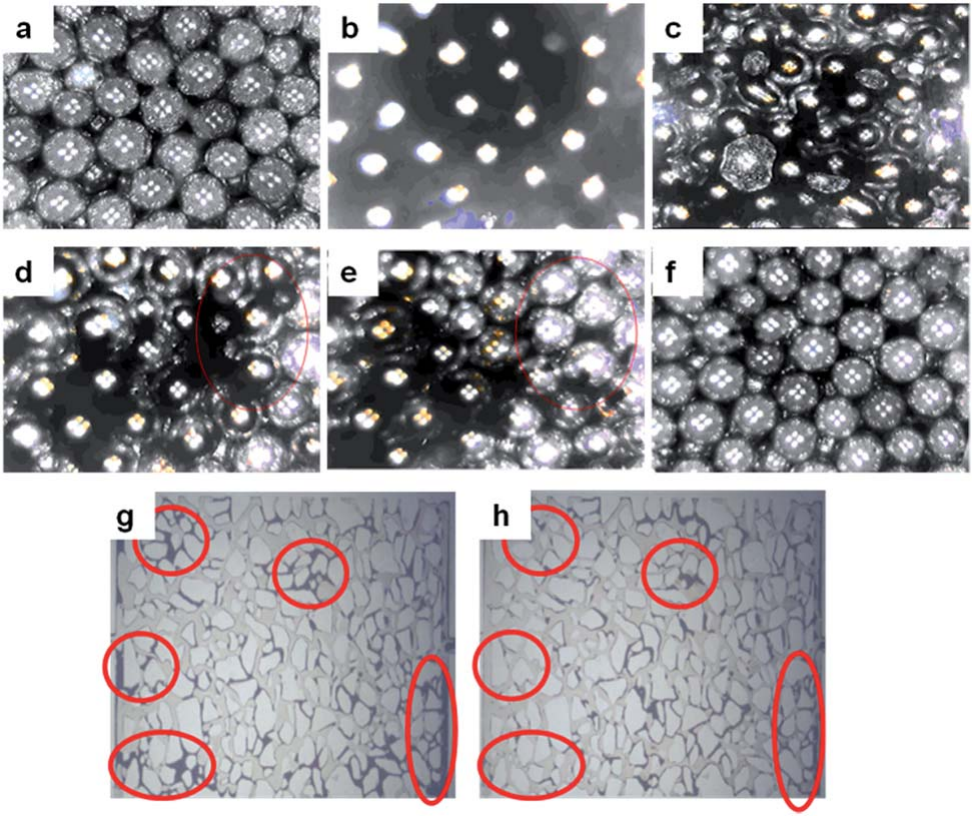
Distribution of oil in the micromodel at different flooding stages: (a) after water saturation; (b) after oil saturation; (c) after water flooding; (d) after HPAM flooding; (e) during B-PPG flooding (f) after B-PPG flooding; residual oil distribution in micro flooding stages (there are still a lot of oil droplets attached to the displacement model after HPAM flooding) (g) and B-PPG flooding (h) (black represents residual oil).

To investigate the oil displacement capability, a micromodel using glass beads is designed and performed on HPAM and B-PPG. Fig. 10 shows the oil distribution in the micromodel on different surfaces of the glass beads after water flooding (Fig. 10c). When HPAM flooding is complete, most of the oil in the large paths is displaced. However, there is still a large amount of oil in the model, especially in the small channels (Fig. 10d, red circle). B-PPG flooding is conducted when no oil can be displaced by HPAM flooding. The residual oil in the large paths is quickly driven out as well as the oil attached on the surface of the glass beads or in the small paths. After B-PPG flooding, almost no residual oil can be detected in the micromodel, demonstrating that B-PPG can displace most of the residual oil. Moreover, by comparing the two pictures from the etched glass micromodel (Fig. 10g and h), we can find that the displacement ability of B-PPG is better than that of HPAM since the residual oil after B-PPG flooding is much less.

**Fig. 11:**
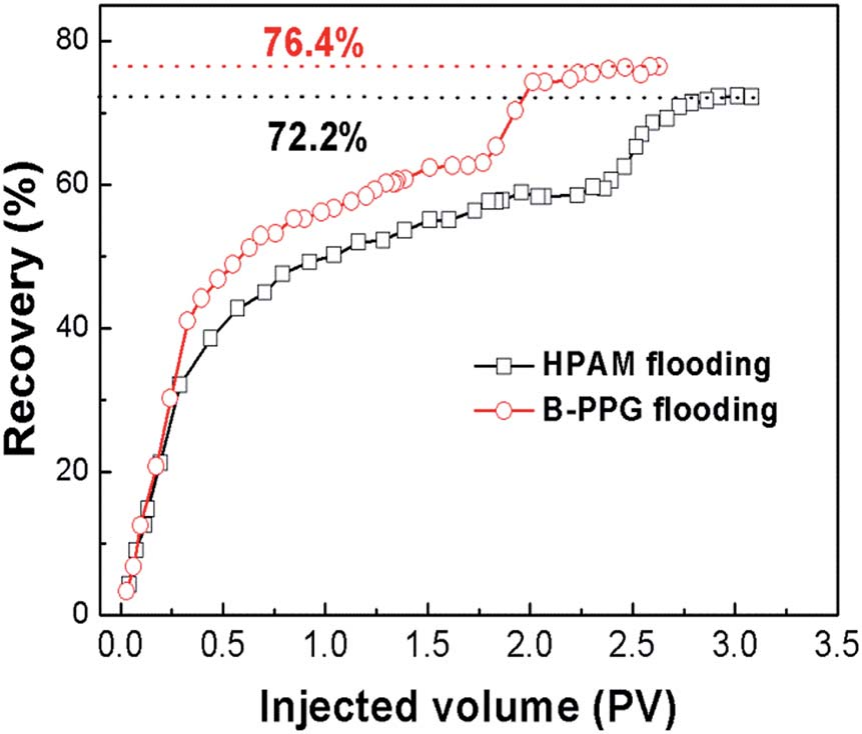
Oil displacement tests of B-PPG (sample 6#) suspension and HPAM solution in the sand pack.

Finally, oil-displacement tests are also conducted. The results in Fig. 11 show that the ultimate recovery of HPAM can achieve a value of 72.25%, while it achieves a value of 76.1% for B-PPG, which is nearly four percentage points higher. The oil-displacement test confidently supports that B-PPG shows higher displacement efficiency than HPAM.

## 4. Conclusion

Laboratory experiments were performed to fully evaluate B-PPG. The suspension of B-PPG at a concentration of 5 g L^−1^ in saline water can achieve a high viscosity of 145 mPa s, and the particles can suspend for a long time with a fairly large suspension volume (above 60%). Meanwhile, B-PPG is capable of elastic deformation and can recover its original shape after leaving small pore-throats. As a result, B-PPG can be used both to improve the sweep efficiency and the dynamic profile control in heterogeneous reservoirs. Compared to HPAM, B-PPG shows a lower temperature and salinity sensitivity, indicating that B-PPG exhibits improved stability under high temperature and salinity. B-PPG also exhibits a dramatic improvement in the long-term thermal resistance with a high post-degradation viscosity. During aging tests, the viscosity of the B-PPG suspension increases first and then decreases slowly. After 12 weeks, the viscosity remains at a value higher than 78 mPa s^−1^. Thus, B-PPG can also be used in secondary polymer flooding. When injected into the reservoir, the B-PPG particles can migrate through the porous media, and the migration is a simultaneous plugging and flooding dynamic process. Besides, B-PPG can significantly create fluid diversion and increase the swept volume in low permeability zones. Moreover, B-PPG is able to displace the residual oil in small channels, leading to a high swept volume and enhanced displacement efficiency. The ultimate recovery of B-PPG in oil-displacement tests is 76.1%, which is nearly four percentage points higher than that of HPAM (72.25%).

## Conflicts of interest

There are no conflicts to declare.

## Supplementary Material

RA-008-C7RA13152F-s001
