# Supplementary material for: Stability, seepage and displacement characteristics of heterogeneous branched-preformed particle gels for enhanced oil recovery
Source: RSC Adv. 2018 Jan 29;8(9):4881–9. doi: 10.1039/c7ra13152f (PMC9078029; doi:10.1039/c7ra13152f)
Supplement: RA-008-C7RA13152F-s001 [file RA-008-C7RA13152F-s001.pdf]

Electronic Supplementary Material (ESI) for RSC Advances.
This journal is © The Royal Society of Chemistry 2018

# SUPPLEMENTARY INFORMATION

# FOR

# Stability, seepage and displacement characteristics of heterogeneous branched-preformed particle gels for enhanced oil recovery

Jiangbo Li[1], Zuming Jiang[2], Yi Wang[1], Zheng Jing[1]* and Guangsu Huang[1]*

[1]College of Polymer Science and Engineering, Sichuan University, Sichuan, Chengdu 610041, China.

[2]Research Institute of exploration & development, Shengli Oilfield, Dongying 257015,China

Correspondence to: Guangsu Huang (E-mail: Guangsu-huang@hotmail.com)

**Synthesis of branched preformed particle gel (B-PPG).** AM (0.84mol) and different amounts of DA (ranges from 0.01 mol to 0.05mol) are dissolved in deionized water (200 ml) and then they are placed in a round-bottomed flask equipped with a reflux condenser, a thermometer and a nitrogen inlet. $N_2$ is purged for at least 30 min to completely remove oxygen. Afterwards, 10 ml of freshly prepared solutions of KPS ($1.5*10^{-4}$ mol) and $NaHSO_3$ ($1.5*10^{-4}$ mol or a little less) are added successively, and the polymerization is carried out in an adiabatic jacket until the highest temperature of the system is reached. Then the flask is transferred to an 85°C water bath for another 2h to ensure the polymerization reacted completely. Finally, the samples in the gel state are carefully removed from the flask. Then they are cut

into pieces, dried, and smashed. Particles of 150~200 mesh are selected for the subsequent tests.

**FTIR spectroscopy.** B-PPGs are purified by solvent extraction method in which alcohol is used as the solvent for about 48hr to remove small molecular compounds thoroughly, and then the resulting samples are recorded in solid state, by KBr pellet method, using a FTIR spectrophotometer (Nicolet IS10 Thermo Fisher Scientific, USA) between 500 and 4000 $cm^{-1}$. PAM is used as a contrast.

**Pyrolytic-gas chromatograph-mass spectra (Py-MS).** The B-PPG sample in the dried powder form is put into a platinic vessel and dropped into the cracking tube freely. Then the pyrolysis products are isolated and analyzed by the GC/MS system (Agilent 6890) through a capillary transfer line, and the pyrolysis temperature is held at 600°C. Chromatograph conditions: A DB25 (30m×0.25mm×0.25μm) chromatograph column is utilized, the carrier gas is He, flow rate of the column is 0.80 mL/min and the spilt ratio is 100:1. The temperature of the column is maintained at 60°C for 3 min and increased to 260°C at 10°C/min. The maximum temperature is also kept for another 5min. Mass spectrometer conditions: An EI source at the temperature of 200°C is selected while the electron energy is 70eV. A mass range among 29～500m/z is scanned.

**Preparation and characterization of the B-PPG.** B-PPG is synthesized by free radical polymerization of AM using DA as cross-linking agents under a redox system at room temperature. When $NaHSO_3$ meets with the initiator KPS, AM polymerization is initiated. The acrylamide radicals can initiate the double bonds in DA to form a main polymer chain. Meanwhile, they can also initiate the methylene carbons next to N atom in DA. Subsequently, long polymer chain radicals are generated when chain propagation occurs on these active carbon free radicals.

Covalent links are formed if coupling termination takes place between these polymer chain radicals. In addition, they can also be terminated by other free radicals to result in branched dangling chains. Moreover, the branched chains can entangled with each other to form physical links. As a consequence, the branched preformed particle gel is successfully constructed. By changing the ratio of DA and AM, B-PPGs of different crosslinking degree can be obtained. A sketch is proposed to illustrate the formation process of B-PPG in Fig.S1.

The FTIR spectra of PAM and B-PPG are shown in Fig.S2 where the peaks located in 3450 ~2800 $cm^{-1}$ are attributed to stretching vibrations of -OH, $-NH_2$ and the hydrogen bonds in the system. The peaks at 1635$cm^{-1}$ and 1608 $cm^{-1}$ are the amide peak for C=O stretching vibration (amide I) and $-NH_2$ shear vibration, respectively. Comparing with the spectrum of B-PPG, there is no apparent shoulder peaks emerged in the pure PAM around 1700$cm^{-1}$, whereas the existence of a characteristic absorption peak of ester carbonyl -C=O at 1719.02$cm^{-1}$ indicates that DA is combined into the product of B-PPG.

Structure of B-PPG is investigated by the pyrolytic-gas chromatograph-mass spectrum (Py-MS). Fig.S2b are the mass spectra of several groups of the molecular or iron fragments under the pyrolysis temperature of 600$^{o}$C which can be analyzed to deduce the pyrolysis process and verify the molecular structure of B-PPG. The strongest peak (m/e=17) is ascribed to the neutral molecule $NH_3$. The peak (m/e=15) belongs to ·CH3 and it demonstrates that DA has participated in the polymerization. The peak (m/e =44) is produced by the amide group linked to the two methylene through α- cleavage which proves the presence of two methylene connecting with each other on the polyacrylamide chain and head-to-head coupling termination in the B-PPG polymerization. This agrees with other scientific workers as they have clearly

pointed out that the carbon chains of PAM contains a proportion of abnormal "head-to-head" linkages which are formed by the recombination of macroradicals[1-2]. Thus, the coupling termination of two radicals on the end of two branched chains can contribute to the covalent cross-linked structure in B-PPG.

The peak (m/z=41) owing to the alkyl nitrile is generated from γH-/α-、α- and σ- cleavage in successively which once again indicates the DA involving in the reaction. And only the imaging molecular structure in Fig.S1 and Fig.S3a is tenable, will the peak (m/e=53) be obtained by the way of γH-/α-、σ-/α-、σ- cleavage in sequence as well as the neutral molecule of acetylene whose peak is on m/e=26. Thus, the molecular structure of B-PPG is evidenced since the radical of methyne which originated from the electron transfer of the methylene carbon beside the N atom initiates the acrylamide monomer polymerization to form a branched chain structure.

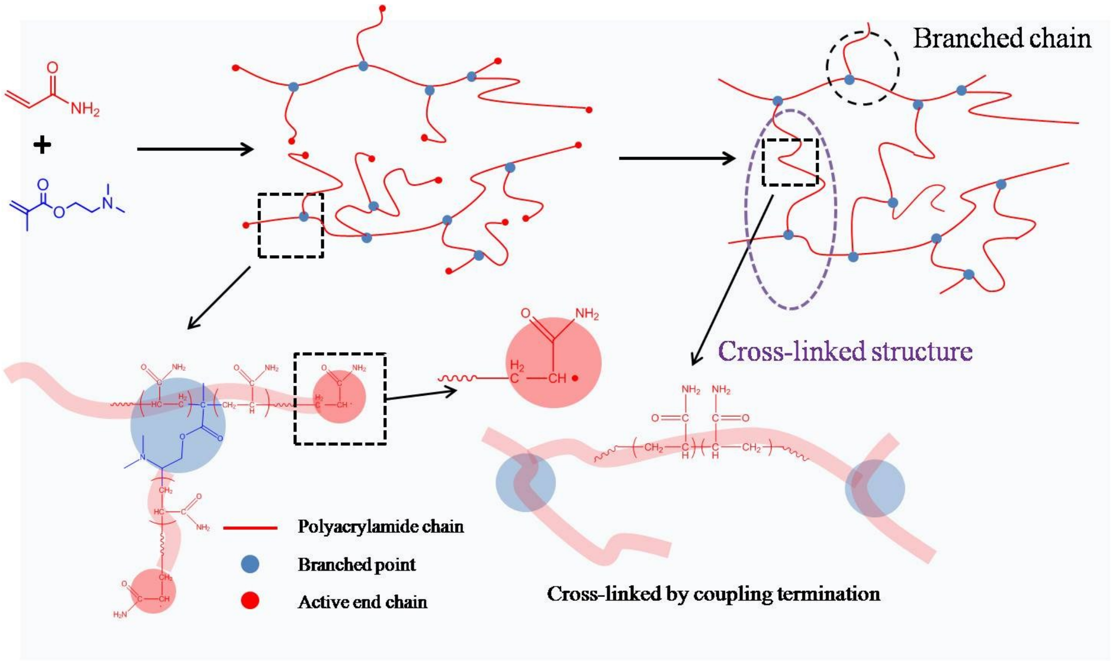


**Fig.S1** A sketch map of the structure for the formation process of branched preformed particle gel (B-PPG) from DA and AM

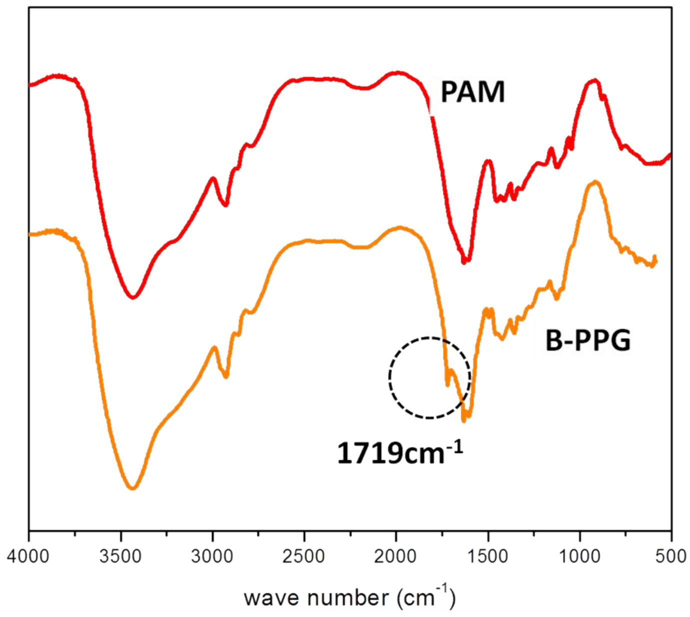


**Fig.S2** FT-IR spectra of PAM and B-PPG.

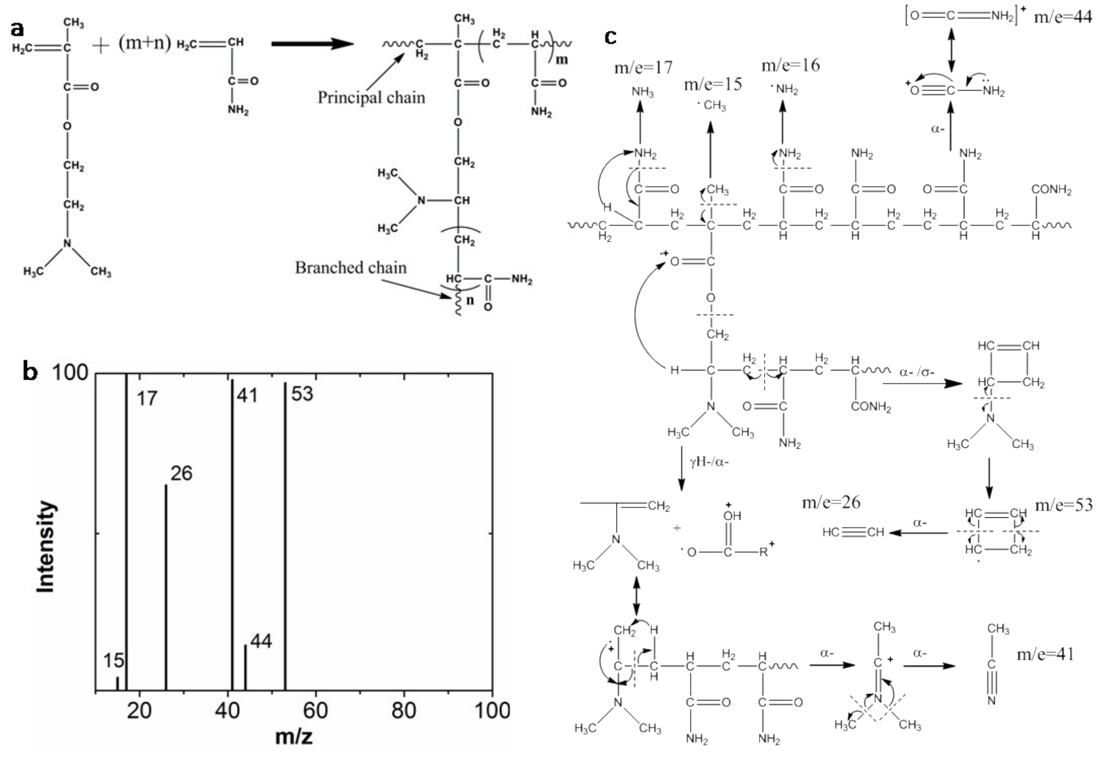


**Fig.S3** (a) Schematic illustration for the reaction mechanism of B-PPG of DA with AM, (b) Mass spectra of B-PPG where several groups of the molecular or iron fragments are displayed, (c) A predicted pyrolysis process for B-PPG.

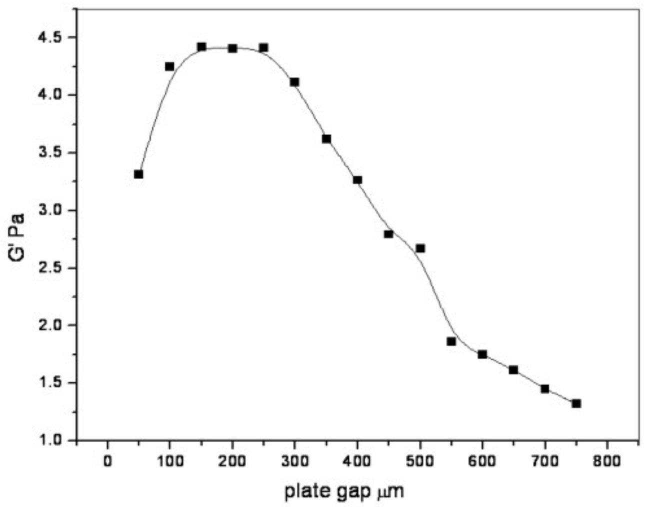


**Fig.S4** The effect of plate gap on the measured G' of B-PPG suspension.

## References

1. Arcus CL, *Journal of the Chemical Society (Resumed)*:2732-2736 (1949).

2. Bao M, Chen Q, Li Y, Jiang G, *Journal of hazardous materials* **184**:105-110. (2010).
